# Generating Datasets for Real-Time Scheduling on 5G New Radio

**DOI:** 10.3390/e25091289

**Published:** 2023-09-02

**Authors:** Xi Jin, Haoxuan Chai, Changqing Xia, Chi Xu

**Affiliations:** 1 Key Laboratory of Networked Control Systems, Chinese Academy of Sciences, Shenyang 110016, China; chaihaoxuan@sia.cn (H.C.); xiachangqing@sia.cn (C.X.); xuchi@sia.cn (C.X.); 2 Shenyang Institute of Automation, Chinese Academy of Sciences, Shenyang 110016, China; 3 Institutes for Robotics and Intelligent Manufacturing, Chinese Academy of Sciences, Shenyang 110169, China; 4 University of Chinese Academy of Sciences, Beijing 100049, China

**Keywords:** 5G new radio, real-time scheduling, dataset, optimization modulo theories, satisfiability modulus theories

## Abstract

A 5G system is an advanced solution for industrial wireless motion control. However, because the scheduling model of 5G new radio (NR) is more complicated than those of other wireless networks, existing real-time scheduling algorithms cannot be used to improve the 5G performance. This results in NR resources not being fully available for industrial systems. Supervised learning has been widely used to solve complicated problems, and its advantages have been demonstrated in multiprocessor scheduling. One of the main reasons why supervised learning has not been used for 5G NR scheduling is the lack of training datasets. Therefore, in this paper, we propose two methods based on optimization modulo theories (OMT) and satisfiability modulo theories (SMT) to generate training datasets for 5G NR scheduling. Our OMT-based method contains fewer variables than existing work so that the Z3 solver can find optimal solutions quickly. To further reduce the solution time, we transform the OMT-based method into an SMT-based method and tighten the search space of SMT based on three theorems and an algorithm. Finally, we evaluate the solution time of our proposed methods and use the generated dataset to train a supervised learning model to solve the 5G NR scheduling problem. The evaluation results indicate that our SMT-based method reduces the solution time by 74.7% compared to existing ones, and the supervised learning algorithm achieves better scheduling performance than other polynomial-time algorithms.

## 1. Introduction

The networking of industrial systems is the foundation of intelligent manufacturing [1]. Networking means connecting a large number of production elements, such as workers, machines, materials, etc., to the information system, and adopting advanced network techniques to implement high-performance communications between these elements [2]. For modern industrial systems, because wired networks are expensive and cannot connect massive elements, wireless networks are the best choice for industrial networking. However, due to performance limitations, traditional industrial wireless networks can only be used to monitor unimportant elements such as environmental information and instrument data of process industry. It has always been difficult to replace wired networks in industrial applications with low-latency requirements. For example, in order to make a robotic arm move smoothly, the latency of its control command to be transmitted from the controller to the actuator must be less than 10 ms [3]. Wired networks can satisfy the latency requirement easily, whereas traditional wireless networks, e.g., WirelessHART and WIA-PA, take about 100 ms [4], which makes them unsuitable for such industrial scenarios.

With the development of 5G, its ultra-reliable and low-latency communication (URLLC) has implemented wireless transmission latency of less than 1 ms [5,6] and makes it possible to support wireless motion control. However, many technical details still need to be improved. For example, to immediately retransmit lost data packets without waiting for an authorization, existing techniques must reserve redundant network resources for retransmissions in advance. However, these redundant reservations waste many resources and severely reduce network capacity. Therefore, in order to adopt 5G in industrial systems, the 5G resources should be scheduled effectively to avoid unnecessary waste.

**Figure 1:**
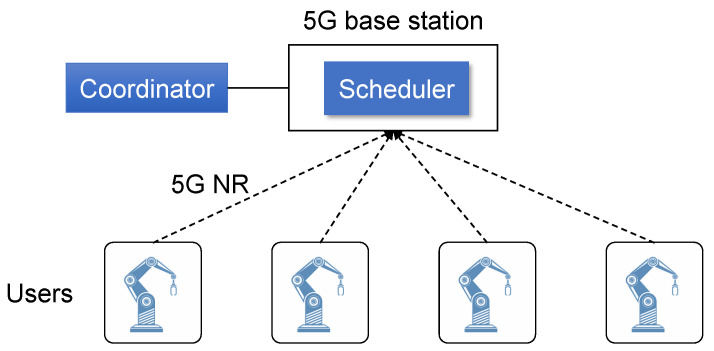
A 5G-based industrial system.

A typical 5G-based motion control system is shown in Figure 1. The coordinator of users is directly connected to a 5G base station, and multiple users are connected to the base station through 5G new radio (NR). The 5G NR protocol is the wireless protocol between users and the base station, i.e., it is the wireless part of 5G. The resources of 5G NR include two dimensions: time and frequency. A real-time scheduling algorithm running on the base station assigns time and frequency resources to data packets and sends the assignment results to the corresponding users. Hence, the real-time scheduling algorithm is the key to making full use of 5G resources under low-latency requirements. In harsh industrial environments, the available wireless resources are very limited, so more effective scheduling algorithms should be used. Although there has been a lot of research on scheduling algorithms for industrial wireless networks, they cannot be used in 5G because multiple subcarrier spacings defined in 5G NR are not considered in other wireless networks. If a scheduling algorithm based on single subcarrier spacing is applied to 5G, its performance will be severely limited.

The real-time scheduling problem of industrial wireless networks with a single subcarrier spacing has been proved to be NP-hard [7]. Only a few simple scheduling problems can find optimal solutions, whereas most scheduling problems cannot even close to optimal solutions. The 5G scheduling problem with multiple subcarrier spacings is more complicated than others. Thus, the scheduling performance is more difficult to guarantee. For such complicated problems, machine learning is a good choice, and its impressive performance has been demonstrated in many problems. In the field of real-time scheduling, some researchers have used supervised learning to solve the task scheduling problem of processors, and the results show that supervised learning can further improve the scheduling performance [8]. However, the work is limited by the training dataset. Because the optimal scheduling results of the systems with many tasks cannot be found, the supervised learning model is only trained by small-size systems. Therefore, creating training datasets is the primary step for supervised-learning-based scheduling.

For 5G NR scheduling, there are no public datasets and related generating algorithms. Therefore, in this paper, we will propose algorithms to generate datasets. Because the solutions in training datasets directly affect the scheduling performance of supervised learning models, to improve the scheduling performance, we will find optimal solutions to constitute datatsets. However, our 5G NR scheduling problem is NP-hard [9]. There are no polynomial-time optimal algorithms. Although some researchers have found optimal scheduling solutions for 5G NR [10,11], their 5G techniques and models are different from ours. Therefore, we propose a method to formalize our 5G NR scheduling problem, and then, based on the formalization, invoke a solver to find optimal solutions. The main contributions of this paper are as follows:

- First, according to the requirements of 5G NR scheduling, we formulate the problem into an optimization–modulo-theories (OMT) problem, which reduces variables by increasing the formalization of the relationship between them.
- Second, to further reduce the difficulty of solving the problem, we transform the OMT problem into an satisfiability–modulo-theories (SMT) problem and then reduce the solution space by tightening the upper and lower bounds of the scheduling objective.
- Third, we use the SMT-based method to generate a large number of optimal solutions and then constitute the dataset for 5G NR scheduling. Compared to existing work, our proposed method reduces the solution time by 74.7%.
- Finally, we use the generated dataset to train a pointer network model, and the evaluation results show that the model can achieve better scheduling performance than existing polynomial-time algorithms.

The rest of the paper is organized as follows: Section 2 reviews related work. Section 3 details the 5G NR scheduling model. Section 4 introduces the corresponding OMT problem. Section 5 transforms the OMT problem into an SMT problem. Section 6 evaluates our methods. Section 7 concludes this paper.

## 2. Related Work

There exist two categories of methods for generating scheduling datasets. The first category involves solving scheduling problems and then compiling the solutions to form a dataset. The second one focuses on expanding existing datasets to make them larger. The following provides introductions to the two categories.

For solving 5G scheduling problems, some methods have been proposed. The work in [12] focused on an ideal scheduling model with fine-grained and flexible frequency resources and proposed an orthogonal frequency-division multiple access-based scheduling method to reduce the impact of low-latency communications on other communications. The work in [13,14] transformed 5G NR scheduling problems into programming problems and then proposed a method based on reinforcement learning to dynamically send packets according to instantaneous requirements. The work in [15] integrated frequency adjustment and resource allocation into one problem and used linear programming and Lagrangian duality to reduce the transmission delay and improve the transmission rate. In order to improve control performance, the work in [10] combined control with 5G and adjusted data transmissions according to current control needs. The work in [11] proposed a scheduling algorithm for multi-user, multiple-input, and multiple-output networks. The algorithm decomposes the problem into multiple sub-problems and runs them on the GPU in parallel, thereby reducing the execution time of the algorithm to 1 ms and realizing fast response to transmission requirements. The work in [16] added the execution time of the proposed scheduling algorithm to its processing process and designed a 5G scheduler based on deep reinforcement learning to improve the system reliability. The work in [17] considered the impact of signaling on scheduling and proposed an easy-to-implement resource reselection mechanism to improve the reliability of 5G vehicle networking. The work in [18] treated signaling as a resource consumption and effectively reduced the transmission latency through system-level optimization. The work in [19] formulated the problem of scheduling broadcast and unicast as a sequential decision problem and proposed a deep reinforcement learning-based algorithm to solve it. The work in [20] proposed a fair request scheduling method to solve the coexistence problem of 5G NR and WiFi. The work in [21] focused on the QoS management for XR traffic in 5G NR and applied a weight-based scheduling policy to ensure traffic QoS. Because the 5G NR scheduling problem addressed in this paper is NP-hard, there is no polynomial-time optimal algorithm for it. To obtain the optimal solution, mathematical solvers must be invoked. However, the execution time of these solvers is excessively long, rendering them infeasible for real industrial applications. As a result, most of the relevant literature proposed non-optimal heuristic methods, leading to a gap between their results and the optimal solution. Although the existing solver-based methods can yield optimal solutions in the absence of time considerations, none have accounted for the non-ideal channel conditions that our problem model focuses on. The problem model in [9] is similar to that in this paper. However, the work in [9] adopted classical real-time theory to solve the 5G NR scheduling problem. There is still a gap between the schedules and the optimal solution. Therefore, in this paper, we will propose two novel methods to obtain optimal solutions, which cannot be obtained in [9].

For expanding the scale of datasets, the generative adversarial network (GAN) is a widely used algorithm at present. GAN is a deep generative neural network proposed by Ian Goodfellow in 2014 [22]. At first, GAN was mainly used for image and text enhancement in the direction of computer vision, but because of its ability to capture the underlying distribution of data, GAN soon was successfully applied to other research fields, such as communication and medical care. The work in [23] used the conditional generic adversary network (CGAN) to generate high-quality tag data under a small amount of seed data. Then, the performance of CGAN was verified on a public dataset, and the evaluation results showed that the dataset generated by CGAN is very similar to the original dataset. The work in [24] proposed deep convolutional generative adversarial networks (DCGAN) to solve the problem of data imbalance in traffic classification. Since DCGAN combines GAN and CNN, it has higher stability than GAN. Although GAN and its extended algorithms are very effective, these algorithms require an existing dataset as a seed. However, for our problem, there are no datasets, and the GAN-based methods cannot be used.

## 3. Problem Model

The network considered in this paper includes a 5G base station and multiple users (as shown in Figure 1). The available frequency bandwidth of the base station is *L*. A data flow can be upstream or downstream. Because the network topology is a star, in the following we ignore the direction of data flows. In the flow set $F=\{f_{1},f_{2},\cdots \}$, all flows have the same period *P*. Each data flow $f_{i}$ generates a packet $v_{i}$ at time j×P, and $v_{i}$ must obtain network resources before its deadline (j+1)×P so that it can be delivered to its destination in time. The packet set *V* includes all packets generated in one period. Since the packet set *V* in different periods are the same, we only consider how to schedule packets in one period. In the other periods, the schedules can be repeated.

**Figure 2:**
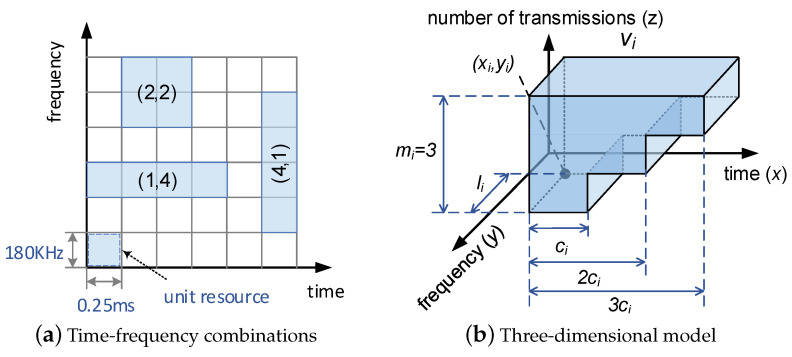
Packet model.

Packet $v_{i}$ is characterized by a three-tuple <$l_{i},c_{i},m_{i}$>, where $l_{i}$ and $c_{i}$ represent the frequency bandwidth and transmission time duration, respectively. The 5G numerology defines three subcarrier spacings for Frequency Range 1, including 180 kHz, 360 kHz, and 720 kHz, and the corresponding time slot lengths are 1 ms, 0.5 ms, and 0.25 ms, respectively. We define 180 kHz and 0.25 ms as the unit bandwidth and the unit time slot. As shown in Figure 2a, each small square represents a unit resource. Then, the three combinations of $l_{i}$ and $c_{i}$ are (1, 4), (2, 2), and (4, 1), all of which contain four resources. A packet can be transmitted within four resources regardless of its length, thus we omit the packet length in our problem model. The variable $m_{i}$ represents the maximum transmission times of packet $v_{i}$. Due to non-ideal channel conditions, retransmissions are necessary to improve reliability. The more transmissions, the higher the reliability of the packet. Different industrial applications have different reliability requirements: the reliability of control-related data should be 99.999% or 99.99999%, whereas the reliability of environmental data can be 99.9%. Thus, different packets need to specify different $m_{i}$. In our network model, the maximum transmission times cannot be greater than *M*, i.e., $\forall v_{i}\in V,1\leq m_{i}\leq M$.

We use a three-dimensional block to denote the resources assigned to a packet (as shown in Figure 2b). Thus, the schedule of packet $v_{i}$ is to specify the coordinates $\left(x_{i},y_{i}\right)$ of the upper-left corner of the base. Then, the resources used for one transmission are <$\left(x_{i},y_{i}\right),\left(x_{i}+c_{i},y_{i}+l_{i}\right)$> (these two diagonal coordinates represent a rectangle of resources); the resources for two transmissions are <$\left(x_{i},y_{i}\right),\left(x_{i}+2\times c_{i},y_{i}+l_{i}\right)$>; for the maximum number of transmissions, the resources are <$\left(x_{i},y_{i}\right),\left(x_{i}+m_{i}\times c_{i},y_{i}+l_{i}\right)$>. During network operation, the packet is first transmitted once on resources <$\left(x_{i},y_{i}\right),\left(x_{i}+c_{i},y_{i}+l_{i}\right)$>. If the transmission fails, the packet continues to occupy resources <$\left(x_{i}+c_{i},y_{i}\right),\left(x_{i}+2\times c_{i},y_{i}+l_{i}\right)$> and is transmitted again; if it succeeds, the subsequent resources can be flexibly used by other packets.

**Figure 3:**
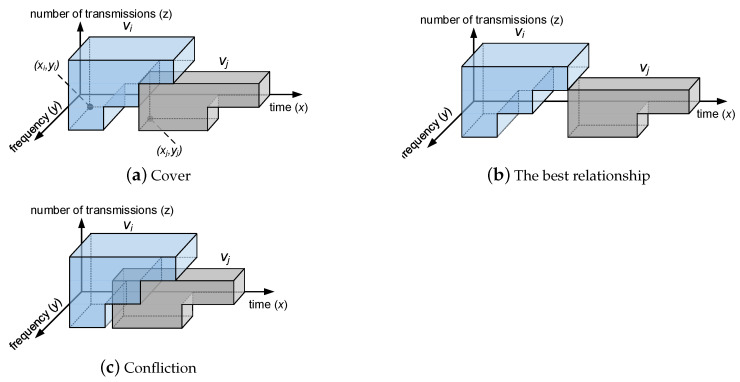
Packet-to-packet relationship.

The flexible-use rule is shown in Figure 3a. If $v_{i}$ covers $v_{j}$, and $v_{i}$ is successfully delivered after two transmissions, then $v_{j}$ can use the resources assigned to $v_{i}$’s third transmission. If $v_{i}$ cannot be delivered after two transmissions, then $v_{j}$ cannot occupy $v_{i}$’s resources and cannot be transmitted. In addition to this rule, when network resources are sufficient, we do not allow any packets to be covered (as shown in Figure 3b). This is because covering $v_{j}$ means $v_{j}$ may not be transmitted and may have to be discarded. Only when the resources are insufficient can the high-reliability packets be allowed to cover the low-reliability packets. Last, but no less important, is that the resources assigned to $v_{i}$ and $v_{j}$ cannot conflict with each other (as shown in Figure 3c). Resource confliction will cause the second transmission of $v_{i}$ to make $v_{j}$ unable to be transmitted. However, the packets with the same number of transmissions are equally important and should not interfere with each other. Based on the above rules, the high-reliability packets have sufficient resources to guarantee their performance, and the low-reliability packets can make full use of idle resources to transmit as much as possible.

## 4. Formulation of the OMT Problem

According to the requirements of 5G NR scheduling, we formulate the problem into an OMT problem, which contains fewer variables than the work in [9]. This simple OMT problem will make the subsequent improvement we introduce in the next section more efficient.

The problem addressed in this paper is as follows: given the packet set *V*, and the available resources L×P, we need to specify the coordinates $\left(x_{i},y_{i}\right)$ for each packet $v_{i}$ so that all packets can be allocated in the available resources, and unimportant packets are covered first if network resources are not sufficient; $x_{i}$ and $y_{i}$ are integer variables, and $r_{i}$ is 0–1 variable. If $r_{i}=1$, then $v_{i}$ is covered by other packets; otherwise $v_{i}$ is not covered. The value ranges are as follows:

(1) $$\forall v_{i}\in V,x_{i}\in \left[0,P-c_{i}\times m_{i}\right),y_{i}\in \left[0,L-l_{i}\right),r_{i}\in \left\{0,1\right\}.$$

When all $x_{i}$ and $y_{i}$ are specified, the corresponding $r_{i}$ can be obtained indirectly. The formulation of $x_{i}$, $y_{i}$, and $r_{i}$ is as follows:

(2) $$\begin{array}{cc} \forall v_{i}\in V, & (\left(r_{i}=1\right)\wedge \underset{\forall v_{j}\in V^{m_{i}+1}\cap ...\cap V^{M}}{\vee }R\left(v_{i},c_{i}\times m_{i},v_{j},c_{j}\times m_{j}\right))\vee \\ \; & (\left(r_{i}=0\right)\wedge \underset{\forall v_{j}\in V^{m_{i}+1}\cap ...\cap V^{M}}{\wedge }R\left(v_{i},c_{i}\times m_{i},v_{j},c_{j}\times m_{j}\right))=true, \end{array}$$

where $V^{m_{i}+1}$ contains the packets with the maximum transmission times of $m_{i}+1$, i.e., $V^{m_{i}+1}=\left\{v_{j}|\forall v_{j}\in V,m_{j}=m_{i}+1\right\}$. Since the packets with higher transmission times can cover $v_{i}$, only these packets are considered in Equation (2); R() is invoked to check if two packets do not cover or conflict with each other, as follows:

(3) $$R\left(v_{i},c_{i}^{\prime },v_{j},c_{j}^{\prime }\right)=\left(x_{i}\geq x_{j}+c_{j}^{\prime }\right)\vee \left(y_{i}\geq y_{j}+l_{j}\right)\vee \left(x_{j}\geq x_{i}+c_{i}^{\prime }\right)\vee \left(y_{j}\geq y_{i}+l_{i}\right),$$

where the parameters $c_{i}^{\prime }$ and $c_{j}^{\prime }$ represent transmission time along the *x*-axis. According to different transmission times, $c_{i}^{\prime }$ and $c_{j}^{\prime }$ are an integer multiple of $c_{i}$ and $c_{j}$. Equation (3) is used to check the relationship between the two rectangles <$\left(x_{i},y_{i}\right),\left(x_{i}+c_{i}^{\prime },y_{i}+l_{i}\right)$> and <$\left(x_{j},y_{j}\right),\left(x_{j}+c_{j}^{\prime },y_{j}+l_{j}\right)$>. Then, the check is true, i.e., two rectangles do not cover or conflict with each other, if one of the following four conditions is true: (1) the bottom side of $v_{i}$ is above the top side of $v_{j}$; (2) the left side of $v_{i}$ is to the right of the right side of $v_{j}$; (3) the top side of $v_{i}$ is below the bottom side of $v_{j}$; (4) the right side of $v_{i}$ is to the left of the left side of $v_{j}$.

In Equation (2), if $v_{i}$ is covered by a packet, i.e., $\underset{\forall v_{j}\in V^{m_{i}+1}\cap \cdots \cap V^{M}}{\vee }R\left(v_{i},c_{i}\times m_{i},v_{j},c_{j}\times m_{j}\right)$ is true, then $r_{i}$ must be equal to 1. Conversely, if $v_{i}$ is not covered by any packet, $\underset{\forall v_{j}\in V^{m_{i}+1}\cap ...\cap V^{M}}{\wedge }R\left(v_{i},c_{i}\times m_{i},v_{j},c_{j}\times m_{j}\right)$ is true and $r_{i}=0$. Hence, we connect $r_{i}$ with $x_{i}$ and $y_{i}$ through R().

This paper focuses on the problem of covering as few packets as possible. Thus, the objective of the problem is to minimize the weighted sum of the number of covered packets, as follows:

(4) $$\min\sum_{\forall m\in [1,M)}\left(w^{m}\times \sum_{\forall v_{i}\in V^{m}}r_{i}\right),$$

where $\omega^{m}$ is the weight of the packets with *m* transmissions, as follows:

(5) $$\forall m\in \left[1,M\right],w^{m}=\left\{\begin{array}{c} 1,\;\;\;\;\;\;\;\;\;\;\;\;\;\;\;\;\;\;\;\;\;\;\;\;if\;\;m=1\\ \sum_{\forall k\in [1,m-1]}\left(w^{k}\times |V^{k}|\right)+1,\;\;others \end{array}\right.$$

When network resources are insufficient, the low-reliability packets are covered first. In other words, if there are low-reliability packets that can be covered, any high-reliability packets cannot be covered. Therefore, the weight of the lowest reliability packet is set to 1, and the other weights $w^{m}$ are greater than the weighted sum of all lower reliability packets.

In addition, the problem must respect the non-conflicting constraint:

(6) $$\forall v_{i}\in V,\forall v_{j}\in V^{m_{i}}\cap ...\cap V^{M},R\left(v_{i},c_{i}\times m_{i},v_{j},c_{j}\times m_{i}\right)=true$$

For $v_{i}$, the other packets are not allowed to use the same resources as $v_{i}$ when they are transmitted the same number of times, as shown in Figure 3c.

Equations (1)–(6) constitute an OMT problem. Based on the formulation, when *V*, *L*, and *P* are given, OMT solvers, e.g., Microsoft Z3, can be used to find the optimal solution. Hence, for different combinations of *V*, *L*, and *P*, the dataset for supervised learning can be obtained by Z3.

## 5. Formulation of the SMT Problem

The Z3 solver, developed by Microsoft Research, contains many advanced theories and methods for solving SMT and OMT problems. Its outstanding performance has been verified in related work. However, for network scheduling, it is difficult to reduce the solution time due to the huge and complicated solution space of the problem. Although Z3 has been used in some network scheduling studies, it is only for very small networks. Even when the scheduling problem is complicated, the network size must be smaller than real networks. Hence, we must reduce the solution time of Z3 to find optimal solutions for larger-scale problems in an acceptable time. Therefore, we have reduced the variables and simplified the problem formulation in Section 4. In this section, we transform the OMT problem into an SMT problem, which further reduces the solution space by tightening the optimization objective of the scheduling problem.

### 5.1. The Basic SMT Problem

The transformed formalization is represented by SMT(A), where *A* corresponds to the objective value of the OMT problem. Hence, we rewrite Equation (4) as follows.

(7) $$\sum_{\forall m\in [1,M)}\left(w^{m}\times \sum_{\forall v_{i}\in V^{m}}r_{i}\right)=A.$$

Equations (1)–(3) and (5)–(7) form the SMT problem SMT(A). The possible values of *A* are brought into SMT(A). Then, the minimum value of *A* that makes SMT(A) satisfiable is the objective value of the original OMT problem, and all $x_{i}$ and $y_{i}$ corresponding to this objective value are the solution of the scheduling problem. Since the order in which the values of *A* are brought into SMT(A) determines how many times the SMT problem is solved, this order is the key to improving the efficiency of solving the problem. In the next subsection, we discuss how to determine the value order of *A*.

### 5.2. Improvement

**Figure 4:**
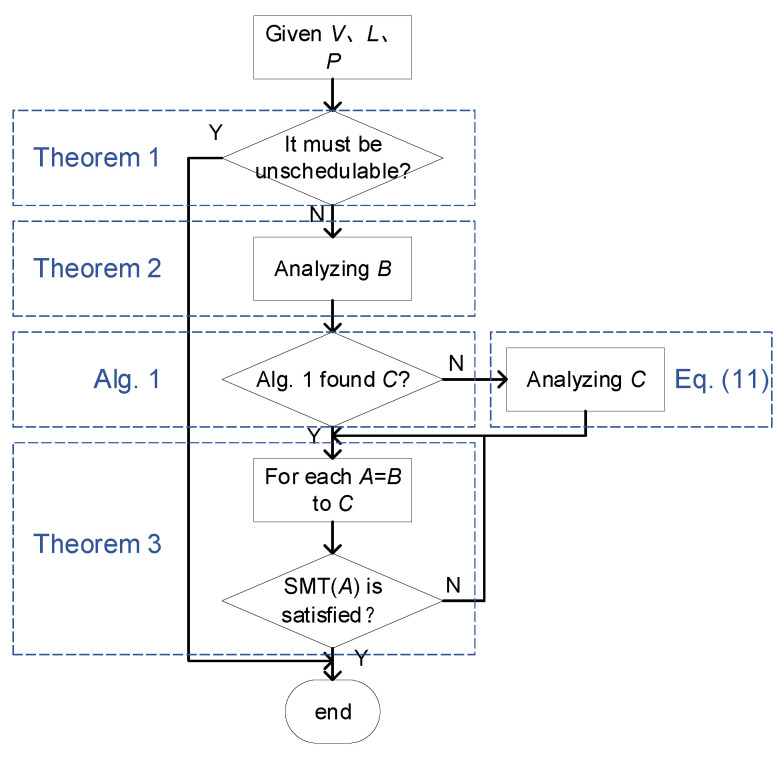
Flow chart of our method.

Our proposed SMT-based method is shown in Figure 4. The method first checks whether the scheduling problem is unschedulable or not based on Theorem 1. If there may be a solution, then we address the following two subproblems: (1) find the lower bound *B* and upper bound *C* of *A* based on Theorem 2, Algorithm 1, and Equation (11); (2) in the range of [B,C], find the minimum value of *A* that makes SMT(A) satisfiable based on Theorem 3. Invoking this method once can generate one solution. By invoking the method multiple times with different inputs, a large number of solutions can be generated and constitute our dataset. In the following, we present our method in detail.

Given a scheduling problem *V*, *L*, and *P*, if the problem satisfied the sufficient condition (Theorem 1), then it must be unschedulable, and the method ends. Otherwise, the method proceeds to the next steps.

**Theorem** **1** (Sufficient condition for unschedulability). *For the scheduling problem V, L, and P, if ∃m∈[1,M], and*

(8) $$\frac{\sum_{\forall v_{i}\in V^{m}}\left(4\times m_{i}\right)}{L\times P}>100\%,$$

*then the problem must be unschedulable.*

**Proof.**  Based on the non-conflicting constraint, the utilization of resource L×P on each slice perpendicular to the Z axis cannot be greater than 100%. The Z axis has a value range of m∈[1,M]. Each *m* corresponds to a slice perpendicular to the Z axis, and $V^{m}$ contains the packets that use this slice resource. Hence, $\frac{\sum_{\forall v_{i}\in V^{m}}\left(4\times m_{i}\right)}{L\times P}$ can be used to calculate the utilization of the slice resource, where 4 represents four resources occupied per transmission. If there exists *m* in [1,M], and the corresponding resource utilization is more than 100%, all packets must not be scheduled without confliction.    □

When there is no cover, *A* should be 0, i.e., 0 must be the lower bound of *A*. However, some 5G NR scheduling problems may not be scheduled without covering. In Theorem 2, we will tighten the lower bound *B* by analyzing the cases that cannot be scheduled without covering, to reduce the number of times to solve SMT.

**Figure 5:**
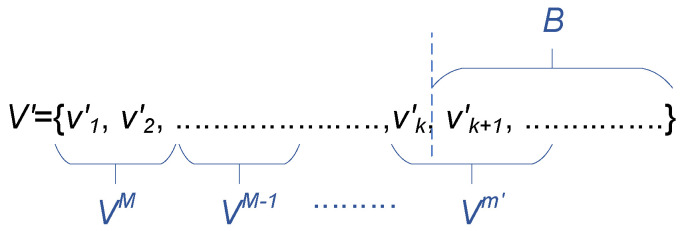
Example of the lower bound *B*.

To prove Theorem 2, we define $V^{\prime }$ as the set containing packets sorted in decreasing order of number of transmissions, i.e., $V^{\prime }=\left\{V^{M},V^{M-1},\cdots ,V^{1}\right\}$, as shown in Figure 5. The packets in each $V^{m}$ (m∈[1,M]) can be in any order because $V^{\prime }$ focuses on the resource usage of packets while the packets in each $V^{m}$ occupy the same amount of resources. We use *k* to represent that, under non-coverable scheduling, the resource requirements for the first *k* packets in $V^{\prime }$ is less than or equal to L×P, and the resource requirements of the first k+1 packets is greater than L×P, i.e.,

(9) $$\sum_{\forall i\in [1,k]}\left(m_{i}^{\prime }\times 4\right)\leq L\times P<\sum_{\forall i\in [1,k+1]}\left(m_{i}^{\prime }\times 4\right),$$

where $V^{m^{\prime }}$ represents the subset containing the *k*th packet. In addition, we extend $w^{0}=0$. Thus, Theorem 2 is as follows.

**Theorem** **2** (Lower bound of *A*). *For the scheduling problem V, L, and P, the lower bound B of A is calculated as follows:*

(10) $$B=\sum_{\forall m\in [1,m^{\prime })}\left(w^{m}\times |V^{m}|\right)+w^{m^{\prime }}\times \left(|V\right|-k-\sum_{\forall m\in [1,m^{\prime })}|V^{m}|)-w^{\left\lfloor \frac{L\times P-\sum_{\forall i\in [1,k]}\left(m_{i}^{\prime }\times 4\right)}{4}\right\rfloor }.$$

**Proof.**  Based on the definition of *k*, in resource L×P, after the first *k* packets are placed, there are no sufficient resources to place the (k+1)th packet. However, perhaps a later packet could be placed. Then, the remaining packets must be covered and considered in the calculation of *B*. The subsequent packets contain all packets in $V^{m^{\prime }-1},\cdots ,V^{1}$, whose weighted objective is $\sum_{\forall m\in [1,m^{\prime })}\left(w^{m}\times |V^{m}|\right)$, and the packets after *k* in $V^{m^{\prime }}$, whose weighted objective is $w^{m^{\prime }}\times \left(|V\right|-k-\sum_{\forall m\in [1,m^{\prime })}|V^{m}|)$. In addition, the weight of the packet that might be placed is $w^{\left\lfloor \frac{L\times P-\sum_{\forall i\in [1,k]}\left(m_{i}^{\prime }\times 4\right)}{4}\right\rfloor }$. Therefore, *B* is the sum of the first two parts, and then minus a placeable packet.    □

Heuristic algorithms can be used to solve the 5G NR scheduling problem. Since the solution of SMT must be better than or equal to those of heuristic algorithms, the solutions of heuristic algorithms can be used as the upper bound *C* of *A*. Our problem is the same as that addressed by the SAC algorithm [9]. Although SAC can be used to determine *C*, it is only suitable for scheduling problems with long times and narrow bandwidth. Therefore, we propose a more applicable algorithm (as shown in Algorithm 1).

The 5G NR scheduling problem is a three-dimensional bin packing problem for irregular objects. The placement space of packets is time–frequency two-dimensional space, whereas the cover and confliction relationship between packets is three-dimensional. Therefore, our heuristic algorithm learns from the classical bottom-left bin packing algorithm [25] and then considers whether the three-dimensional relationship between packets satisfies the scheduling constraints. For each object, the classical bottom-left algorithm selects the bottommost leftmost position that can be placed without confliction. The two-dimensional space of the bottom-left algorithm is placed from bottom to top, whereas our scheduling method should be placed on the left first because packet models occupy resources from left to right. Thus, we adopt a left-top strategy. For each packet in *V*, the left-top strategy searches for available places in L×P first in the leftmost column from top to bottom, then in the second column from top to bottom, and so on. The first place where the packet does not conflict with the other packets in L×P×M is its final place. Hence, the time complexity of the simple strategy is $O\left(|V|LPLPM\right)=O\left(n^{6}\right)$. In order to reduce the solution time, our proposed heuristic algorithm simplifies the search process and reduces the time complexity to $O\left(LP+|V|LPM\right)=O\left(n^{4}\right)$. The pseudocode of our algorithm is shown in Algorithm 1.
**Algorithm** **1 **Packet placement algorithm.**Input: ***V*, *L*, *P***Output: **obj or FAIL1:obj=0; x=0, y=L−1; ∀x,y,z,S[x][y][z]=0;2:sort the packets of *V* in descending order of the number of transmissions, and store them in $V^{\prime \prime }$ in order;3:**for **i=1 to $|V^{\prime \prime }|$ **do**4:     **if** $LT(v_{i}^{\prime \prime },S,x,y,0)$ returns $\left(x_{i}^{\prime \prime },y_{i}^{\prime \prime }\right)$ **then**5:        $\left(x_{i}^{\prime \prime },y_{i}^{\prime \prime }\right)$ is the place of $v_{i}^{\prime \prime }$, then update S[][][];6:        $V^{\prime \prime }=V^{\prime \prime }-\left\{v_{i}^{\prime \prime }\right\}$;7:        $\left(y_{i}^{\prime \prime }==0\right)?\left(x=x_{i}^{\prime \prime }+1,y=L-1\right):\left(x=x_{i}^{\prime \prime },y=y_{i}^{\prime \prime }-1\right)$;8:**if** $V^{\prime \prime }$ is an empty set **then**9:     **return** 0;10:**else**11:   x=0;y=L−1;12:**for** each $v_{i}^{\prime \prime }$ in $V^{\prime \prime }$ **do**13:   **if** $LT(v_{i}^{\prime \prime },S,x,y,1)$ returns $\left(x_{i}^{\prime \prime },y_{i}^{\prime \prime }\right)$ **then**14:       $\left(x_{i}^{\prime \prime },y_{i}^{\prime \prime }\right)$ is the place of $v_{i}^{\prime \prime }$, then update S[][][];15:       $\left(y_{i}^{\prime \prime }==0\right)?\left(x=x_{i}^{\prime \prime }+1,y=L-1\right):\left(x=x_{i}^{\prime \prime },y=y_{i}^{\prime \prime }-1\right)$;16:       $obj=obj+w^{m^{\prime \prime }}$;17:   **else**18:     **return** FAIL;19**return** obj;

Algorithm 1 first initializes the variables (line 1): obj is the objective value (Equation (4)), i.e., the upper bound *C*; (x,y) are the coordinates that our left-top strategy is checking, and the initial value is (0,L−1). The array S[x][y][z] marks whether resources are occupied by packets. If S[x][y][z]=1, the corresponding resource is occupied. To reduce resource fragmentations, we sort all packets of *V* in descending order of the number of transmissions. If multiple packets have the same number of transmissions, the packet with long transmission time is queued first. Then, the sorted packets are contained in the set $V^{\prime \prime }$ (line 2) and also placed in the same order (line 3). Function $LT(v_{i}^{\prime \prime },S,x,y,0)$ represents that, given the resource occupation S[][][], the left-top strategy searches for available places for $v_{i}^{\prime \prime }$ starting from the coordinates (x,y), and the last parameter means that no packets are allowed to be covered. If the last parameter is 1, then packets can be covered. The coordinates $\left(x_{i}^{\prime \prime },y_{i}^{\prime \prime }\right)$ returned by $LT(v_{i}^{\prime \prime },S,x,y,0)$ is the place of $v_{i}^{\prime \prime }$. Then, the corresponding S[][][] is updated (line 5), $v_{i}^{\prime \prime }$ is removed from $V^{\prime \prime }$ (line 6), and (x,y) is modified to search for the next place (line 7). If $LT(v_{i}^{\prime \prime },S,x,y,0)$ does not return available coordinates, $v_{i}^{\prime \prime }$ cannot be placed without covering and remains in $V^{\prime \prime }$. After all packets of $V^{\prime \prime }$ are processed, if $V^{\prime \prime }$ is empty (line 8), then the algorithm finishes, and the objective value is zero because no packets are covered (line 9); otherwise, the remaining packets in $V^{\prime \prime }$ are placed again (line 11). The remaining packets can be covered, and $LT(v_{i}^{\prime \prime },S,x,y,1)$ is invoked to search for an available place (line 13). If there is an available place, then the packet is placed, and S[][][], *x*, *y*, and obj are updated (lines 14–16); otherwise, the algorithm cannot find an available schedule for the problem (lines 17–18). Finally, the objective value obj is returned (line 19), which is the upper bound *C* of *A*.

Because our heuristic algorithm is not an optimal algorithm, for some solvable problems, it cannot find feasible solutions. Therefore, when our heuristic algorithm cannot return the upper bound, we calculate one:

(11) $$C=\sum_{\forall m\in [1,M)}\left(w^{m}\times |V^{m}|\right).$$

Equation (11) means that all packets are covered except those with the maximum number of transmissions. This is the worst case. The value of *A* must not be greater than Equation (11). Therefore, the worst case *C* must be the searching boundary of *A*.

Based on the above theorems and algorithm, we can obtain the lower bound *B* and upper bound *C* of *A*. However, although the values of *A* can be ordered in the range [B,C], the satisfiability of SMT(*A*) is not monotonic with respect to the order of *A* (Theorem 3). Therefore, to avoid missing the minimum value of *A*, we must check the values one by one from *B* to *C* until a value satisfies SMT(*A*). The value is the objective value of our problem. Since *B* is a tight bound, the objective value can be found after a few verifications. Even so, the solution time of the SMT-based method is still less than that of the OMT method. The comparison between them is shown in Section 6.

**Theorem** **3** (Non-monotonicity). *The satisfiability of SMT(A) is not monotonic with respect to the order of A.*

**Proof.**  If SMT(*n*) (n>0) is satisfiable, SMT(n−1) and SMT(n+1) may or may not be satisfiable. We list four cases to illustrate that there can be any relationship among SMT(n−1), SMT(*n*), and SMT(n+1).

(1) SMT(*n*) is satisfiable, and SMT(n+1) is satisfiable. Based on SMT(*n*), if an uncovered packet with $m_{i}=1$ can be placed under other packets, then SMT(n+1) is satisfiable.
(2) SMT(*n*) is satisfiable, and SMT(n+1) is not satisfiable. Based on SMT(*n*), if there is no uncovered packet with $m_{i}=1$, then SMT(n+1) must not be satisfiable.
(3) SMT(*n*) is satisfiable, and SMT(n−1) is satisfiable. After SMT(*n*) is satisfied, if available resources are sufficient to make a covered packet with $m_{i}=1$ uncovered, then SMT(n−1) is satisfiable.
(4) SMT(*n*) is satisfiable, and SMT(n−1) is not satisfiable. After SMT(*n*) is satisfied, if there are no resources available, and no packet can be covered anymore, then SMT(n−1) cannot be satisfiable.

□

By combining Theorem 1 (sufficient condition for unschedulability), Theorem 2 (lower bound of *A*), Algorithm 1 (upper bound of *A*), Equation (11) (upper bound of *A*), and Theorem 3 (non-monotonicity), the 5G NR scheduling problem can be solved quickly by Z3. The time complexities are as follows: checking Theorems 1 and 2 are $O\left(M|V|\right)=O\left(n^{2}\right)$ and O(|V|)=O(n), respectively; searching the value of *A* is $O\left(\sum_{\forall m\in [1,M)}\left(w^{m}\times |V^{m}|\right)\right)=O\left(n\right)$; Algorithm 1 is $O(n^{4})$; Equation (11) is O(n). Therefore, excluding Z3, the time complexity of our proposed method is $O(n^{4})$.

## 6. Evaluation

Generating the dataset includes two parts. The first part involves generating scheduling problems (Section 6.1), and the second part involves solving these problems and generating the dataset of 5G NR scheduling based on our proposed method (Section 6.2). After that, we train a supervised learning model to demonstrate the effectiveness of the dataset (Section 6.3). All the methods run on a Dell Precision T5820 Workstation.

### 6.1. Generating Scheduling Problems

We generate scheduling problems based on the following parameters: *L*, *P*, *V*, and $\forall v_{i}\in V$, <$l_{i},c_{i},m_{i}$>. The values of *L*, *P*, and the number of packets in *V* can be configured by users of our method. To make the dataset uniformly distributed, we use Uunifast [26] to generate <$l_{i},c_{i},m_{i}$> for all packets. The values of $\left(l_{i},c_{i}\right)$ are selected in (1,4), (2,2), and (4,1), and $m_{i}$ is in the range [1,M], where *M* can be specified according to the reliability requirements of industrial applications.

**Figure 6:**
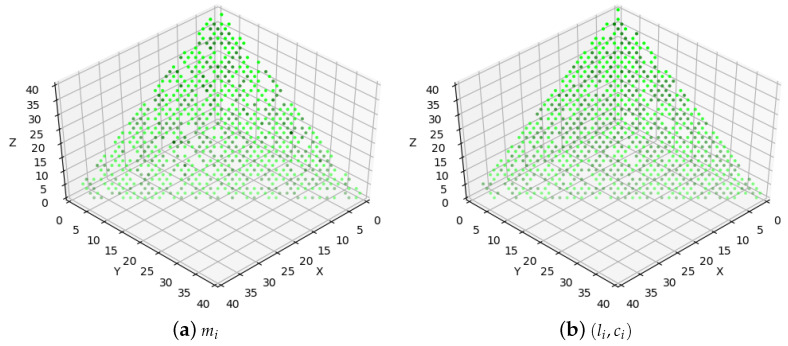
Distribution of scheduling problems.

Figure 6 shows the parameter distribution of the scheduling problems. We generate 10,000 scheduling problems under M=3, |V|=40. Uunifast is invoked to assign the values of $m_{i}$ and $\left(l_{i},c_{i}\right)$. In Figure 6a, for each scheduling problem, the number of packets with $m_{i}=1$ (or 2, 3) is the *x* (or *y*, *z*) coordinate. Thus, a scheduling problem corresponds to a point. The darker the color of the point, the more scheduling problems coincide at this point. Figure 6b is the distribution of $\left(l_{i},c_{i}\right)$. Similarly, the number of packets with (1,4) (or (2,2), (4,1)) is the *x* (or *y*, *z*) coordinate. Figure 6 shows that the scheduling problems are uniformly distributed.

### 6.2. Solving Scheduling Problems

Based on the generated scheduling problems, we evaluate our methods: the OMT-based method (abbreviated OMT) of Section 4 and the SMT-based method (abbreviated SMT) of Section 5. The method used for comparison is the OMT-based method in [9] (abbreviated OMT_original), as its model is similar to ours. All scheduling problems are solved by Microsoft singled-thread Z3. We use the parameter setting <|V|,M,T,L,P> to represent a group of scheduling problems, where *T* is the time limitation of Z3, and |V| corresponds to the network scale. The solution time of Z3 is difficult to estimate. Even for the scheduling problems with the same parameters, some can be solved within seconds, whereas others cannot find a feasible solution within hours. To improve efficiency, if the solution time exceeds *T* minutes, the scheduling problem is discarded, and a new scheduling problem is generated. Since OMT and OMT_original cannot generate scheduling results for complicated problems, we will first evaluate the three methods with simple scheduling problems in Section 6.2.1 and then illustrate the scalability of SMT in Section 6.2.2.

#### 6.2.1. Performance Comparison

We compare the three methods under <[20,30],4,2,7,40> and modify these parameters in turn to evaluate our method across as many system configurations as possible. Based on our experience, setting *T* to 2 allows our SMT method to obtain solutions for the majority of scheduling problems. In the following subsection, we will discuss the impact of different values of *T* on the solutions.

**Figure 7:**
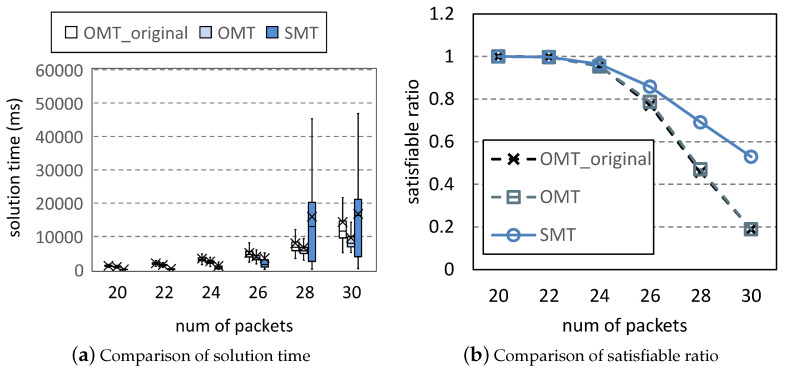
Scheduling problems under <[20,30],4,2,7,40>.

**Figure 8:**
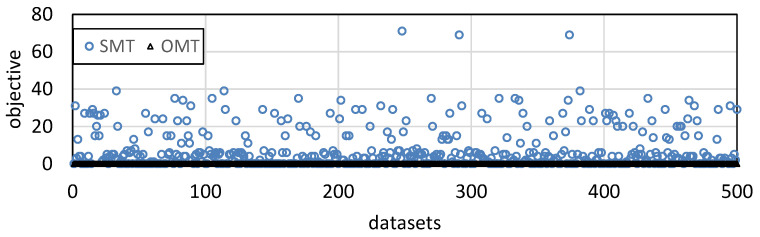
Distribution of objective values.

Figure 7 is the comparison of the solution time and satisfiable ratio of the three methods. *Satisfiable ratio* is the percentage of scheduling problems for which a method can find an optimal solution. For each column, 10,000 scheduling problems are generated by Uunifast. In Figure 7a, the solution time increases with the number of packets. In the first four columns, OMT is better than OMT_original because OMT introduces fewer variables and significantly reduces the search space; SMT has the best solution time because the determination of the objective value simplifies the search for solutions. In the last two columns, OMT and OMT_original can only find solutions for simple problems under the time limitation, whereas SMT can solve more complicated problems. Thus, the solution time of SMT is higher than the others. This is also shown in the last two columns of Figure 7b, in which the satisfiable ratios of OMT and OMT_original significantly decrease. These two methods can no longer solve the problems that SMT needs to consume a lot of time to solve. In Figure 8, we plot the distribution of the objective values of SMT and OMT under <30,4,2,7,40>. The objective values of OMT are all zero. In an acceptable time, OMT cannot solve any problems with covering, whereas SMT can still solve complicated problems to make solutions show diversity. We run 16 Z3 threads on our workstation to solve 10,000 problems under parameter <30,4,2,7,40>. OMT_original takes 3.92 days, whereas OMT and SMT take 1.36 days and 0.99 days, respectively. Compared to OMT_original, SMT reduces the solution time by 74.7%.

**Figure 9:**
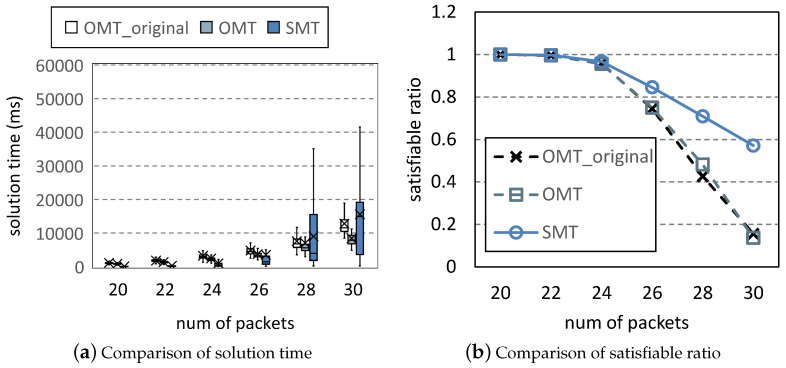
Comparison under varying *T*.

In Figure 9, we change the time limitation *T* to 10. Although there is a slight difference between Figure 7 and Figure 9, the difference is more due to the randomness of Uunifast. The added time has little effect on searching solutions. Since the solution time of solvers grows exponentially with the problem’s complexity, for such scheduling problems, the added time is not enough to solve more complicated problems. Therefore, in our evaluation, the time limitation of 2 can effectively avoid time consumption.

**Figure 10:**
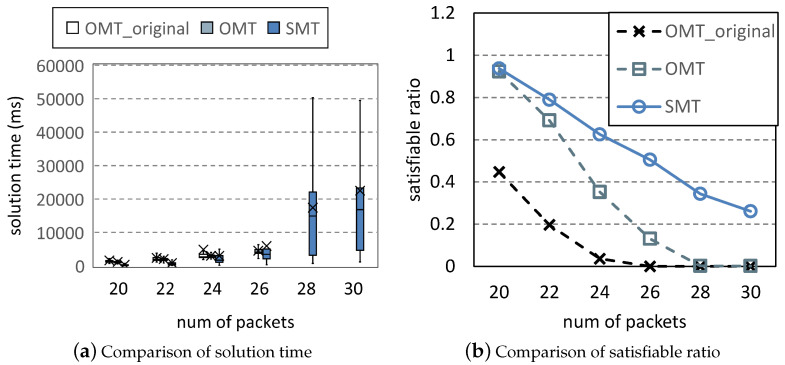
Comparison under varying *M*.

In Figure 10, we increase the maximum number of transmissions from 4 to 5. Since packets require more resources, it is more difficult to place them in L×P. Hence, the satisfiable ratios of all methods are reduced. OMT and OMT_original cannot find any solutions when the number of packets is large. Although the solution time of SMT increases, it can still maintain the satisfiable ratio of 34% when the other methods fail to solve problems.

**Figure 11:**
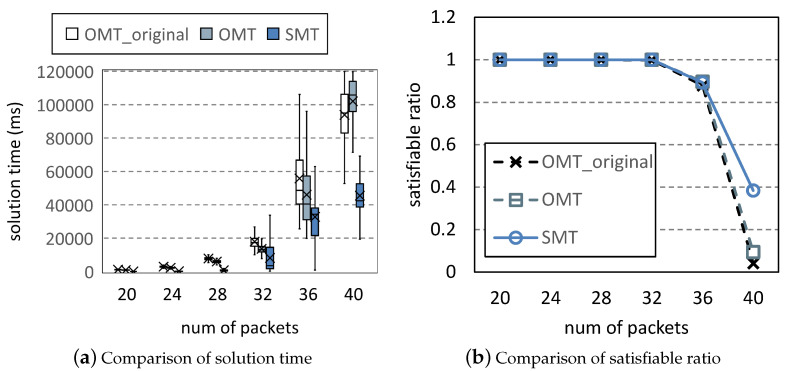
Comparison under varying *L*.

Figure 11 changes the frequency bandwidth from 7 to 10, and at the same time increases the number of packets to 40 to use these increased resources. As the number of packets increases, the solution time increases and the satisfiable ratio decreases. OMT and OMT_original consume a lot of time and can only solve a small amount of scheduling problems. The time growth trend of SMT is slower than those of the other methods. Compared to Figure 7 and Figure 11 increases resources by 42.9%, but reduces the solution time to 4.4%.

**Figure 12:**
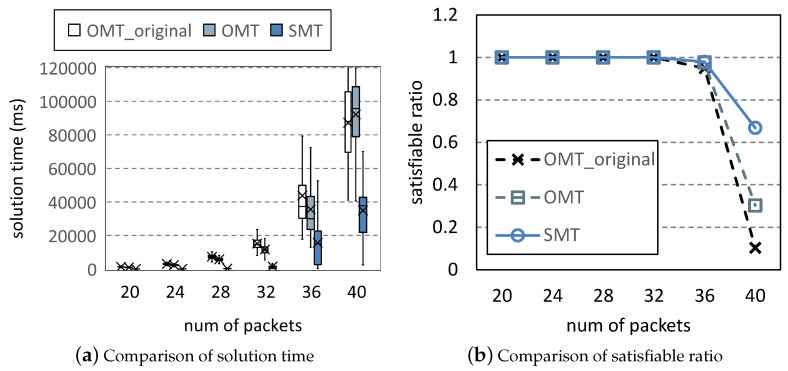
Comparison under varying *P*.

Figure 12 increases the period to 60. Figure 12 is similar to Figure 11. Thus, increasing time and bandwidth both have similar effects on scheduling problems, and for the larger problems, the performance improvement of SMT is more significant.

#### 6.2.2. Scalability of the SMT-Based Method

**Figure 13:**
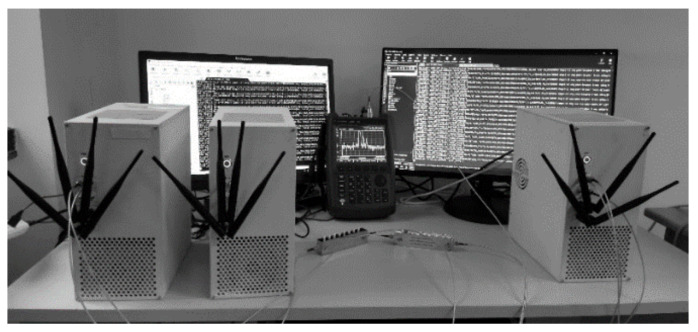
Test platform.

The following evaluation increases the complexity of scheduling problems; OMT_original and OMT cannot solve them, and the trend of the satisfiable ratio is similar to those in the above evaluation. Thus, only the solution time of SMT is shown in the following. In order to make our evaluation closer to actual systems, we set parameters based on a 5G testbed, which is shown in Figure 13. The 5G testbed contains several 5G-URLLC devices [27], operating in the 2.4 GHz unlicensed industrial scientific and medical band. After running the testbed for 4 h, we found that the average packet loss rate for a single transmission is 22%. This means that if a packet is transmitted *M* times, its reliability will be 1–22${\%}^{M}$. In order to ensure a reliability of 99.999%, we set the maximum number of transmissions to at least 8.

**Figure 14:**
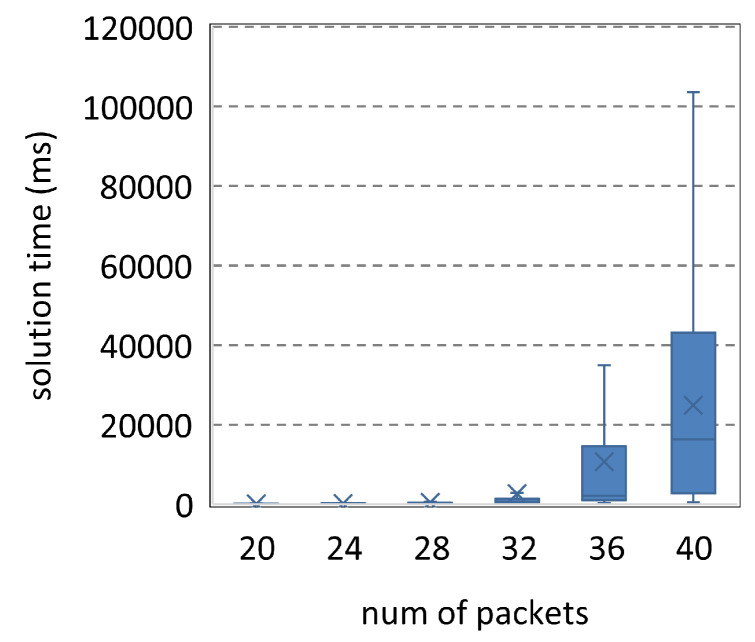
Parameters <[20,40],9,2,24,40>.

**Figure 15:**
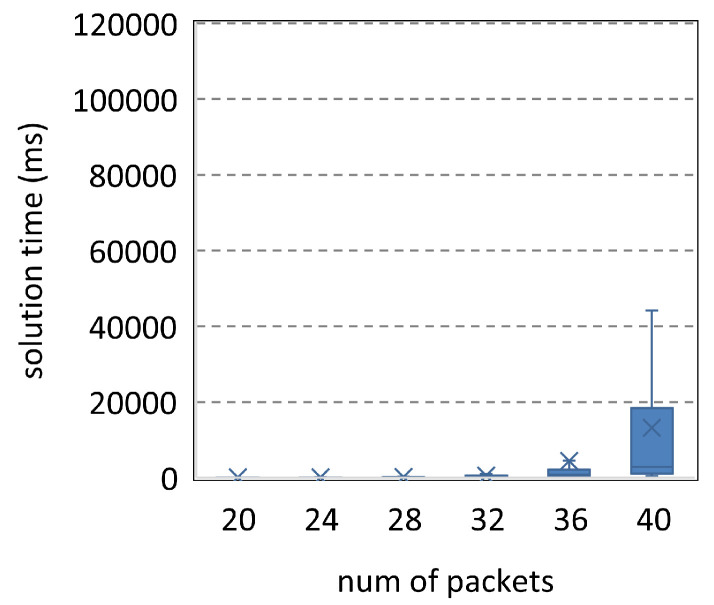
Parameters <[20,40],8,2,24,40>.

**Figure 16:**
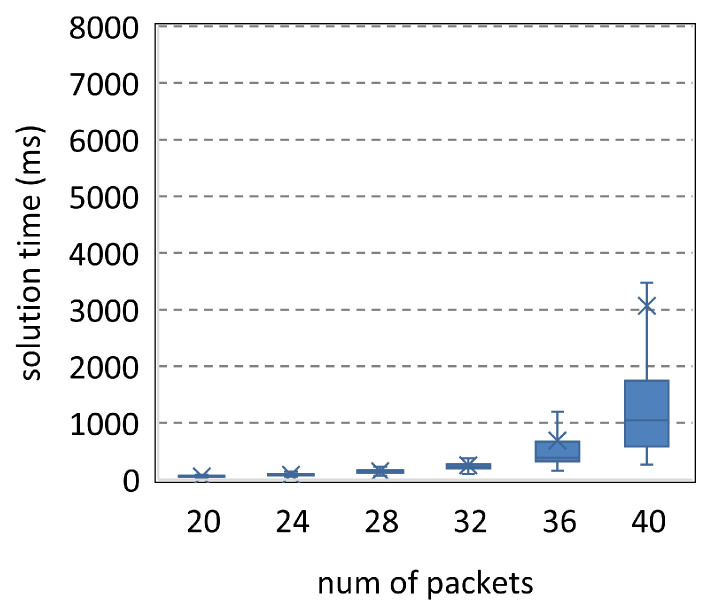
Parameters <[20,40],9,2,32,40>.

**Figure 17:**
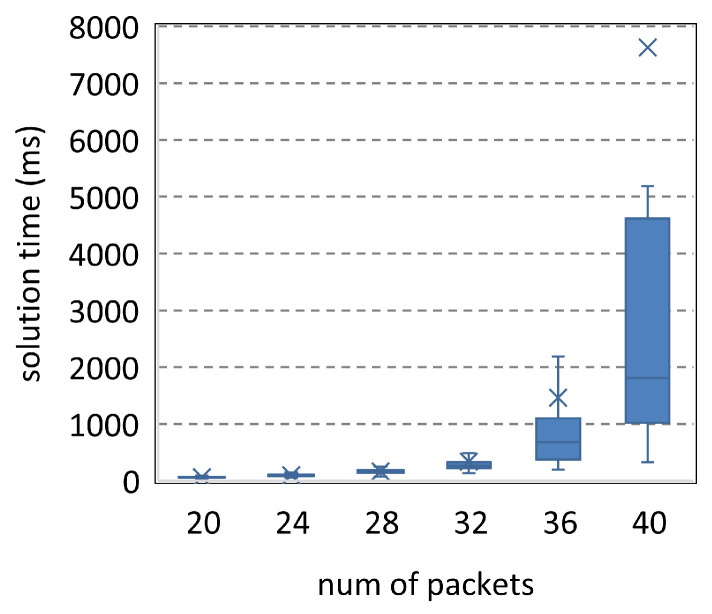
Parameters <[20,40],9,2,24,50>.

The parameters in Figure 14 are <[20,40],9,2,24,40>. The solution time of SMT increases significantly with the number of packets. Figure 15 reduces the number of transmissions to 8. Thus, the solution time decreases. Compared to Figure 14, Figure 16 and Figure 17 increase the resources by about a third to a quarter, while their ordinates are only one-fifteenth of that in Figure 14. Therefore, SMT can be used to solve the complicated scheduling problems, and more resources will significantly reduce the solution time.

The dataset for the 5G NR scheduling problem generated in this paper can be downloaded from this website (accessed on 29 August 2023): https://xijinnetwork.github.io/5GNRDS.html.

### 6.3. Pointer Networks

**Figure 18:**
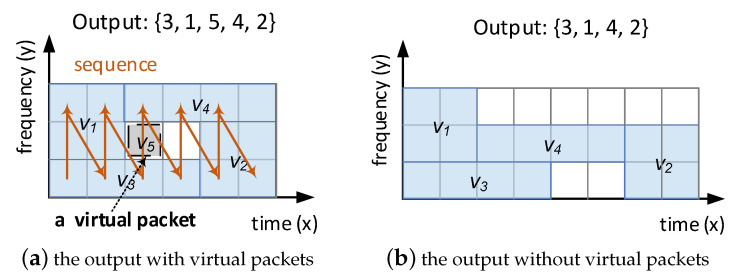
Output sequence.

To demonstrate the effectiveness of the generated dataset, we use it to train a supervised learning model—pointer networks [28]. Pointer networks are a type of neural network that can be used to schedule software tasks [8]. The input of pointer networks is the sequence of task parameters, and the output is the sequence of task indexes, which represent the execution order of tasks. Thus, for 5G NR scheduling, similarly, the input is the sequence of packet parameters {<$l_{1},c_{1},m_{1}>,<l_{2},c_{2},m_{2}$>,...}. However, the output sequence has to be changed, because packet scheduling is different from task scheduling. In task scheduling, multiple processors cannot be combined to serve a task. Thus, when multiple processors are available, no matter which processor the task is executed on, the execution result is not affected. However, in packet scheduling, multiple adjacent frequencies can be combined to serve a packet. Therefore, the execution result depends not only on the order in which the packets are executed, but also on how the frequencies are assigned. To address the problem, we define the output sequence to be first along the frequency dimension and then along the time dimension (as shown in Figure 18a). Furthermore, we add some virtual packets to align resource usage. A virtual packet occupies only one resource, i.e., its parameters are <1,1,1>. Thus, the output sequence of Figure 18a is {3,1,5,4,2}, where $v_{5}$ is a virtual packet. If $v_{5}$ is not added, the output sequence {3,1,4,2} corresponds to Figure 18b, which cannot be reconstructed to the original solution as shown in Figure 18a.

**Figure 19:**
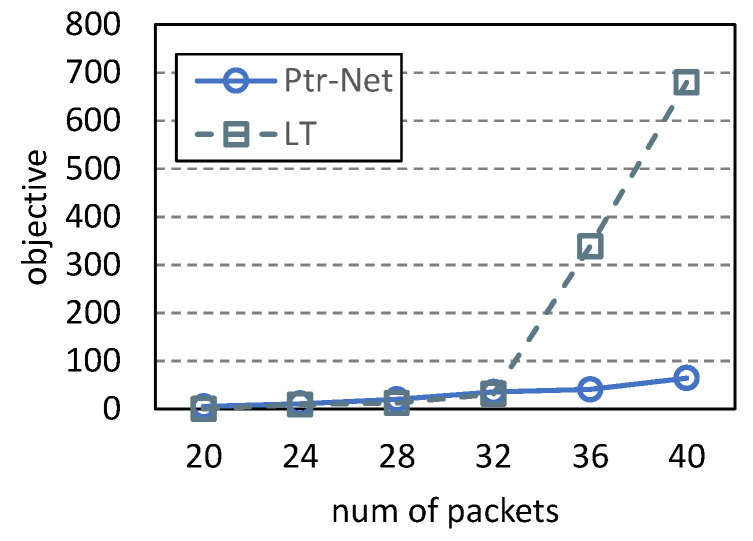
Comparison of objective values.

**Figure 20:**
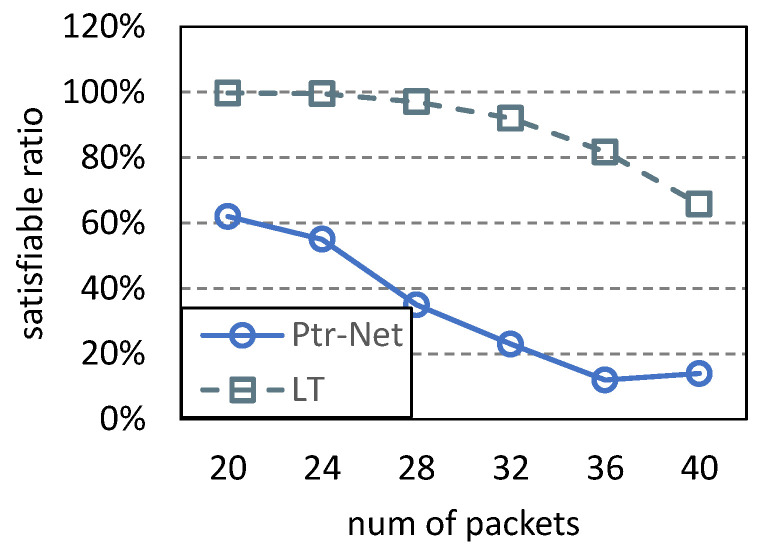
Comparison of satisfiable ratios.

Based on the input/output structure, we reorganize the generated dataset. Then, the neural architecture of pointer networks is set up as follows: (1) Encoder/Decoder: LSTM (long short-term memory) with 512 hidden units; (2) Batch size: 256; (3) Optimizer: Adam; (4) Loss function: sparse categorical cross-entropy; (5) Learning rate: cosine annealing; (6) Epochs: 50. Since, in related work, there is no polynomial-time algorithm for the 5G NR scheduling problem, we compare the pointer network (abbreviated Ptr-Net) with the left-top strategy (abbreviated LT) as shown in Algorithm 1. The comparison results are shown in Figure 19 and Figure 20. As shown in Figure 19, the pointer network can achieve a better objective value than LT. As the number of packets increases, the objective value of LT increases rapidly, while that of Ptr-Net remains almost stable. This is because the coverage strategy employed by LT is greedy, and more packets are covered. However, the objective value of Ptr-Net is not affected by the number of packets because the pointer network can learn the optimal execution order of packets. Because LT covers more packets, its satisfiable ratio is higher, as shown in Figure 20. The pointer network used in this paper is traditional. If more novel neural network models are proposed, training them based on our datasets would lead to improved performance. Since this paper focuses on generating datasets, we will conduct in-depth research on supervised learning in the future.

## 7. Conclusions

The 5G NR scheduling is the key to improving the 5G performance. However, there is still a gap between the solution obtained by scheduling algorithms and the optimal solution. Recently, related work has introduced supervised learning to real-time multiprocessor systems and indicated that supervised learning can fill the performance gap. However, for the 5G NR scheduling problem, due to the lack of training datasets, supervised learning cannot be used. Therefore, first, we formulate the 5G NR scheduling problem into an OMT problem, which uses fewer variables than existing work. Although the OMT problem can be solved by solver Z3, the solution time is unacceptable. Then, to reduce the solution time, we transform the OMT problem into an SMT problem and propose three theorems and an algorithm to reduce the search space. Finally, we evaluate our methods. The evaluation results indicate that, compared to existing work, our proposed method reduces the solution time by 74.7%, and the supervised learning model trained on the generated dataset is effective to schedule packets on 5G NR. In the future, we will design a new neural architecture to improve the performance of 5G NR scheduling.

## Data Availability

Not applicable.
